# Atorvastatin attenuates intermittent hypoxia-induced myocardial oxidative stress in a mouse obstructive sleep apnea model

**DOI:** 10.18632/aging.203339

**Published:** 2021-07-21

**Authors:** Xiao-Bin Zhang, Hui-Juan Cheng, Ya-Ting Yuan, Yan Chen, Yi-Yuan Chen, Kam Yu Chiu, Hui-Qing Zeng

**Affiliations:** 1 Department of Pulmonary and Critical Care Medicine, Zhongshan Hospital, Xiamen University, Teaching Hospital of Fujian Medical University, Xiamen, Fujian Province, People’s Republic of China; 2 Department of Medical Affairs, Zhongshan Hospital, Xiamen University, Teaching Hospital of Fujian Medical University, Xiamen, Fujian Province, People’s Republic of China

**Keywords:** atorvastatin, intermittent hypoxia, myocardial, oxidative stress, apoptosis

## Abstract

Chronic intermittent hypoxia (CIH), a hallmark of obstructive sleep apnea (OSA), is associated with various cardiovascular diseases. In the present study, we assessed the effect of the lipid reducing agent atorvastatin on CIH-induced myocardial oxidative stress and apoptosis in a mouse OSA model. Forty-eight C57BL/6J mice were evenly divided among normoxia + vehicle, normoxia + atorvastatin, CIH + vehicle, and CIH + atorvastatin groups. CIH consisted of a hypoxia-reoxygenation cycle in which oxygen concentrations fluctuated from 21% to 6% and back over two minutes for 8 hours each day (30 events/hour). CIH exposure continued for 12 weeks. Atorvastatin (5 mg/kg) was administered from week 6 through the end of the experiment. CIH increased malondialdehyde levels and decreased superoxide dismutase activity, total antioxidant capacity, and nuclear factor erythroid 2-related factor 2 levels in cardiac tissue, indicating a reduction in antioxidant activity. Atorvastatin significantly reversed those effects (*p* < 0.05). CIH also increased B-cell lymphoma 2-associated protein X and cleaved caspased-3 levels as well as the myocardial apoptotic rate, as indicated by terminal deoxynucleotidyl transferase dUTP nick-end labeling. Atorvastatin had no effect on those changes (*p* > 0.05). Thus, atorvastatin administration exerts antioxidant but not anti-apoptotic effects after CIH and may therefore have therapeutic potential in OSA patients with cardiovascular comorbidities.

## INTRODUCTION

Statins, which inhibit 3-Hydroxy-3-methylglutaryl coenzyme A reductase (HMG-CoA) activity, are widely used in the treatment of dyslipidemia and to decrease the severity of other cardiovascular events in patients with coronary heart disease. More recently, pleiotropic protective effects of statins, including antioxidant, pro-apoptosis, and anti-inflammatory effects in vascular tissue, have been described [1–3]. These findings indicate that statins may have additional therapeutic uses.

Obstructive sleep apnea (OSA), which is characterized by recurrent partial or complete collapse of the upper airway during sleep, is characterized by pathophysiological chronic intermittent hypoxia (CIH) and sleep fragmentation [4]. The overall prevalence of OSA among the general population is about 6–17% [5], and the incidence and mortality of cardio-cerebrovascular diseases is significantly increased among OSA patients. Our previous study confirmed that, independent of obesity, OSA is closely associated with dyslipidemia and hypertension [6]. In another of our previous studies, high renal apoptotic rates were detected after CIH exposure [7], while oxidative stress was increased in CIH-treated mice [8]. Treatments that reduce oxidative stress and apoptosis might therefore be useful therapies for those with OSA. Atorvastatin has been identified as a potential treatment for OSA. Deng and co-workers [9] showed that atorvastatin can reduce CIH-induced hippocampal neuron damage through the toll-like receptor 4 (TLR4) signaling pathway. Atorvastatin was also found to prevent deleterious IH-induced cardiovascular effects, including increased systolic pressure, changes in carotid artery compliance and endothelial function, increased NADPH, and decreased aortic superoxide dismutase activity [10]. Myocardial damage caused by CIH was also ameliorated by administration of atorvastatin [11].

Because OSA is a chronic disease, associated intermittent hypoxia in affected individuals should also be chronic. However, previous studies have used relatively short CIH durations. In this study, we examined the effects of 12 consecutive weeks of CIH on myocardial oxidative stress and apoptosis, as well as the effects of atorvastatin on these physiological changes, in a mouse model to more accurately assess the pathophysiological features of OSA.

## RESULTS

### Body weight and serum biochemical parameters between groups

**Figure 1:**
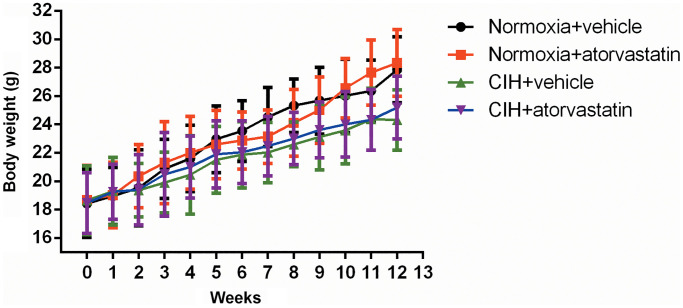
**Mean body weight by group at indicated time points.** Two-way ANOVA for repeated measures was used to examine interaction effects between week and CIH; the results showed that this interaction was not statistically significant [F = 0.494, *p* > 0.05]. Body weights tended to be lower in the CIH group during the last 3 weeks than in the normoxia group, but this difference did not reach statistical significance (CIH + vehicle vs. normoxia + vehicle: 23.58 ± 2.35 vs. 26.01 ± 2.58 at week 10, 24.36 ± 2.18 vs. 26.35 ± 2.18 at week 11, and 24.32 ± 2.13 vs. 27.84 ± 2.32 at week 12, *p* > 0.05).

As shown in Figure 1, body weights in the CIH group tended to be lower than those of normoxia group mice in the last three weeks of the experiment, but this trend did not reach statistical significance (*p* > 0.05). There were no significant differences in any lipid profiles between the normoxia + vehicle and CIH + vehicle groups (all *p* > 0.05, Table 1). Atorvastatin significantly decreased serum TC levels both in the normoxia and CIH conditions (all *p* < 0.05), but no other lipid profile differences were observed among the groups (Table 1). ALT and AST levels tended to be slightly higher in groups that received atorvastatin (Normoxia + atorvastatin and CIH + atorvastatin) than in those that didn’t (Normoxia + vehicle and CIH + vehicle), but these trends also did not reach statistical significance (*p* > 0.05, Table 1).

**Table 1:** Serum lipid profiles in mice by group (*n* = 12 in each group).

**Group**	**Normoxia + vehicle**	**Normoxia + atorvastatin**	**CIH + vehicle**	**CIH + atorvastatin**
TC (mmol/L)	3.45 ± 1.35	2.35 ± 0.98^*^	3.98 ± 1.37	2.68 ± 1.05^#^
TG (mmol/L)	0.98 ± 0.46	1.23 ± 0.78	1.18 ± 0.39	0.95 ± 0.55
HDL (mmol/L)	1.35 ± 0.79	1.45 ± 0.54	1.46 ± 0.79	1.32 ± 0.62
LDL (mmol/L)	2.30 ± 0.95	2.54 ± 1.27	1.95 ± 1.48	2.05 ± 0.89
ALT (IU/L)	32.52 ± 8.42	40.35 ± 10.68	39.69 ± 11.23	43.62 ± 12.35
AST (IU/L)	36.78 ± 10.37	44.32 ± 9.35	43.27 ± 8.92	47.35 ± 11.66

^*^p < 0.05 when compared with normoxia + vehicle group; ^#^*p* < 0.05 when compared with the CIH + vehicle group.

Abbreviations: TC: total cholesterol; TG: triglyceride; HDL: high-density lipoprotein; LDL: low- density lipoprotein; ALT: alanine aminotransferase; AST: aspartate aminotransferase.

### Myocardial histopathological examination

**Figure 2:**
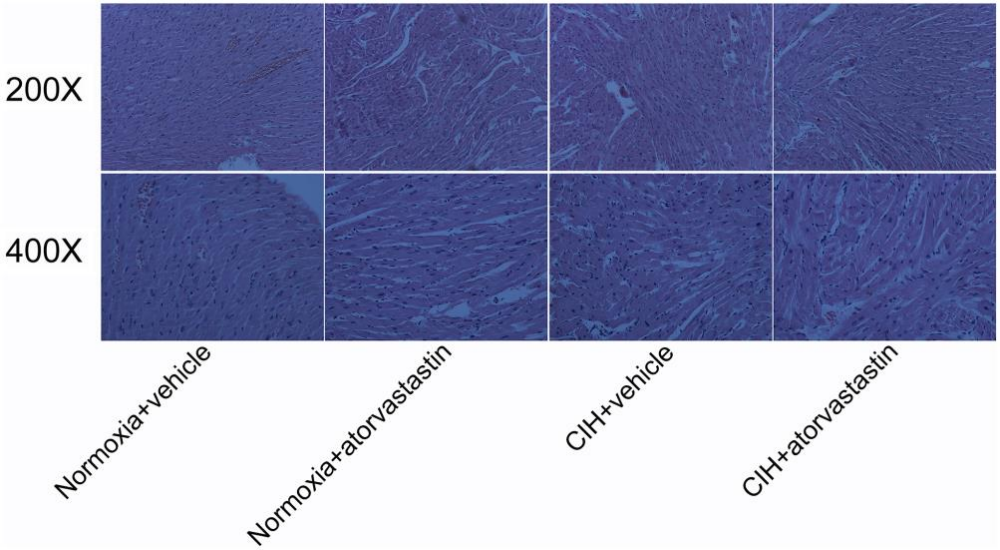
**Histopathological examination of myocardial tissue.** No abnormal architectural features were observed in myocardial tissue using a microscope at different magnifications in any group.

HE staining was performed to observe histopathological changes in myocardial architecture. No abnormal myocardial tissue features were observed in any of the groups under 100× or 400× magnification (Figure 2).

### Atorvastatin decreased CIH-induced myocardial oxidative stress

**Figure 3:**
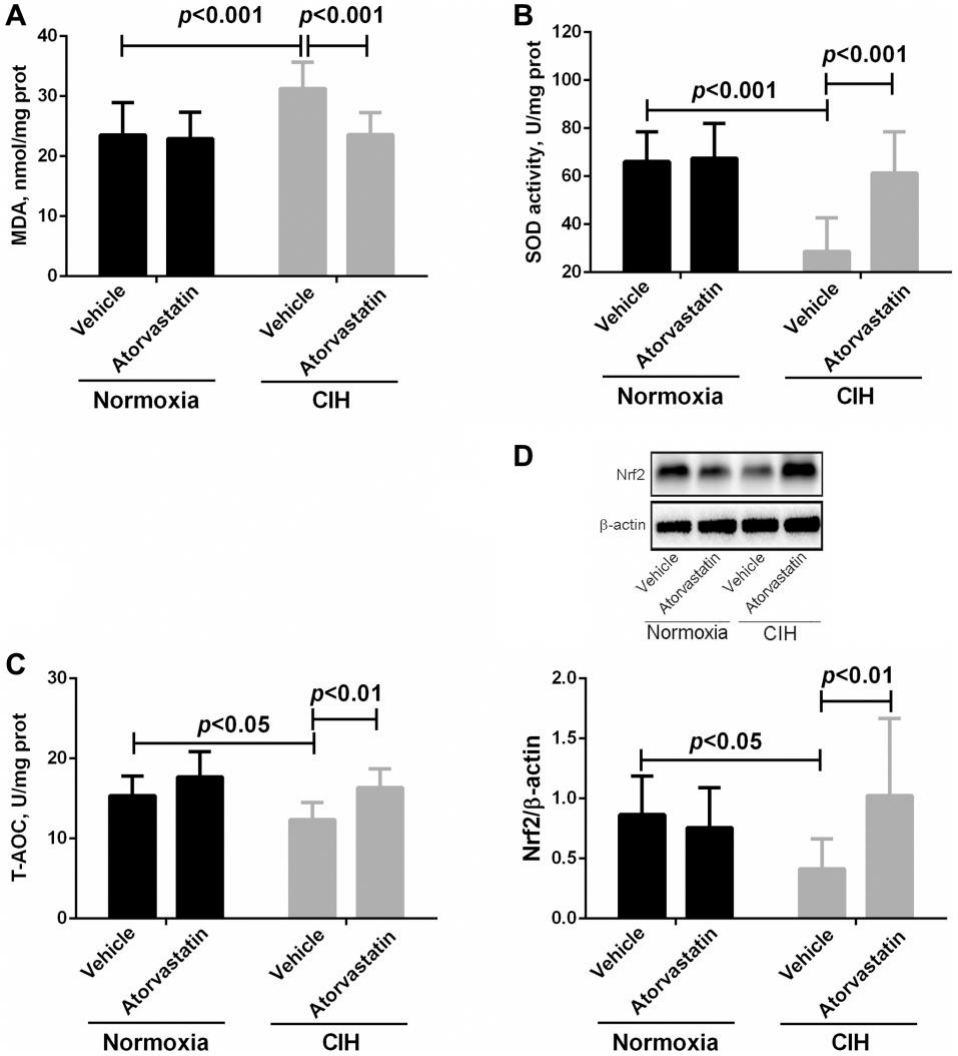
**Differences in oxidative stress markers in myocardial tissue between groups.** In comparison to normoxia + vehicle group, myocardial tissue of mice in the CIH + vehicle group had higher MDA levels (23.54 ± 5.39 vs. 31.25 ± 4.44, *p* < 0.001) and lower SOD activity (66.10 ± 12.34 vs. 28.68 ± 13.91, *p* < 0.001) and T-AOC levels (15.35 ± 2.45 vs. 12.35 ± 2.15, *p* < 0.05). Atorvastatin significantly decreased MDA levels (23.58 ± 3.68 in CIH + atorvastatin group vs. 31.25 ± 4.44 in CIH + vehicle group, *p* < 0.001) and increased SOD activity (61.35 ± 17.13 in CIH + atorvastatin group vs. 28.68 ± 13.91 in CIH + vehicle group, *p* < 0.001) and T-AOC levels (16.35 ± 2.37 in CIH + atorvastatin group vs. 12.35 ± 2.15 in CIH + vehicle group, *p* < 0.01) in IH-induced mice (**A**, **B**, and **C**). Nrf2 levels were lower in the CIH + vehicle group than in the normoxia + vehicle group (0.41 ± 0.25 vs. 0.87 ± 0.32, *p* < 0.05), and atorvastatin treatment increased Nrf2 levels in CIH mice (1.02 ± 0.64 vs. 0.41 ± 0.25, *p* < 0.01) (**D**).

Two-way ANOVAs indicated that there were no interaction effects between CIH and atorvastatin treatment (*p* > 0.05). However, the main effects of CIH and atorvastatin on oxidative stress variables were significant (all *p* < 0.05, Table 2). CIH significantly increased MDA levels and decreased SOD activity and T-AOC levels (all *p* < 0.05). Furthermore, atorvastatin treatment significantly reversed these changes in MDA, SOD activity, and T-AOC in the CIH condition (all *p* < 0.05), but not in the normoxia group (all *p* > 0.05, Figure 3A–3C). Western blot results showed that Nrf2 expression was decreased (*p* < 0.05) in CIH group mice and that atorvastatin administration significantly reversed this change (*p* < 0.05, Figure 3D).

**Table 2:** Main and interaction effects of CIH and atorvastatin on oxidative stress parameters.

	**MDA**		**SOD activity**		**T-AOC**		**Nrf2**	
	**F**	***p***	**F**	***p***	**F**	***p***	**F**	***p***
CIH	10.30	<0.001	27.03	<0.001	8.67	<0.05	4.41	<0.05
Atorvastatin	10.11	<0.001	16.55	<0.001	18.49	<0.01	9.05	<0.01
Interaction	1.35	>0.05	0.98	>0.05	1.27	>0.05	0.56	>0.05

Abbreviations: MDA: malondialdehyde; SOD: superoxide dismutase; T-AOC: total antioxidant capacity; Nrf2: nuclear factor erythroid 2-related factor 2.

### Atorvastatin did not affect CIH-induced myocardial apoptosis

**Figure 4:**
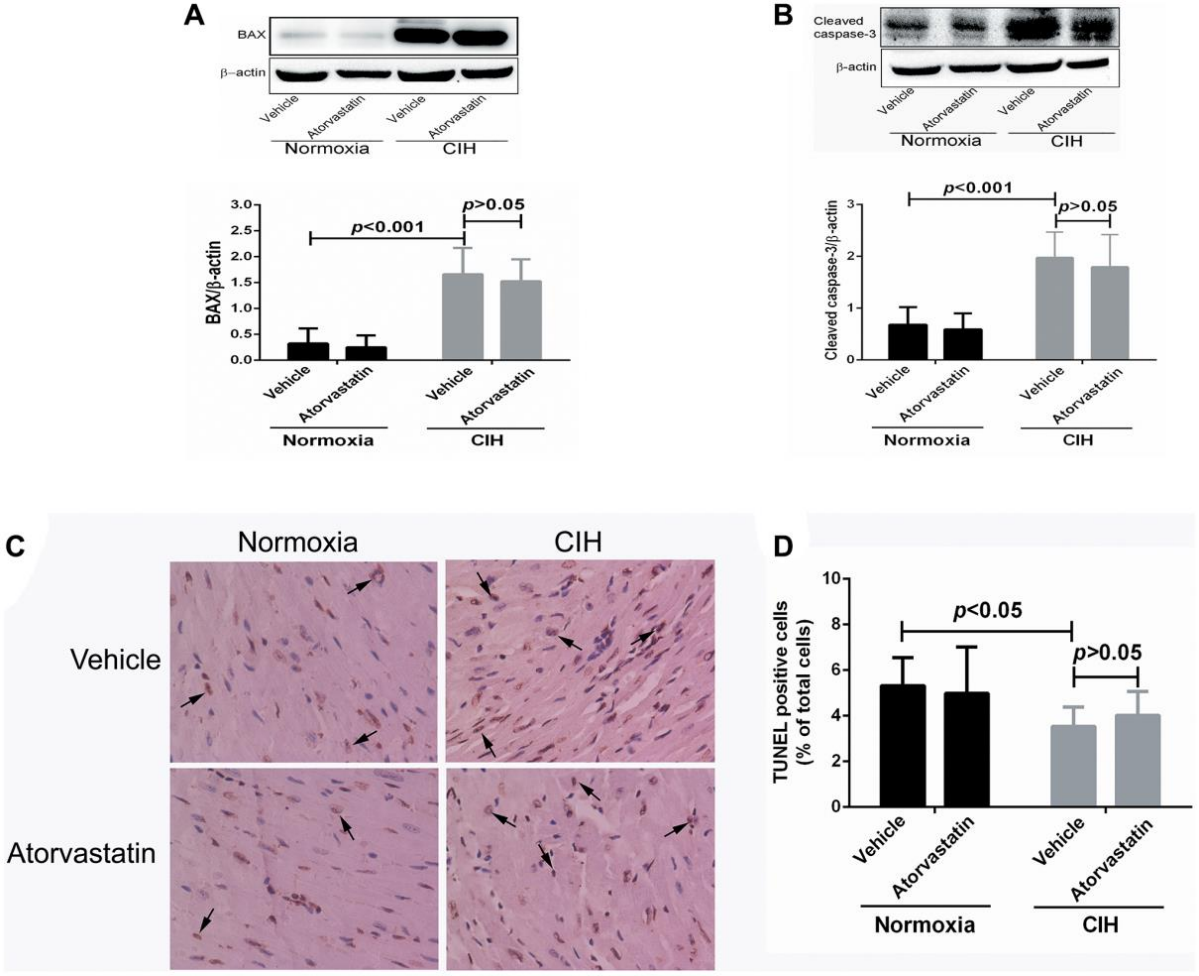
**Myocardial apoptosis by group.** BAX (0.32 + 0.29 vs. 1.66 + 0.51, *p* < 0.001) and cleaved caspase-3 expression (0.68 + 0.34 vs. 1.98 + 0.49, *p* < 0.001) were higher in CIH + vehicle group mice than in normoxia + vehicle group mice (**A** and **B**). CIH exposure also increase the number of TUNEL positive cells in the heart (3.54 + 0.85 in normoxia + vehicle group, 5.32 + 1.23 in CIH + vehicle group, *p* < 0.05) (**C** and **D**). However, atorvastatin administration did not affect CIH-induced apoptosis levels (4.98 + 2.03 in CIH + atorvastatin group, 5.32 + 1.23 in CIH + vehicle group, *p* > 0.05).

Two-way ANOVAs revealed statistically significant main effects of CIH on apoptotic variables (all *p* < 0.05), but no significant effects for atorvastatin treatment (all *p* > 0.05). No interaction effects between CIH and atorvastatin were identified (*p* > 0.05) (Table 3). CIH-induced increases in BAX and Cleaved caspase-3 levels remained unchanged after mice received atorvastatin treatment (all *p* > 0.05, Figure 4A and 4B). TUNEL staining results showed that CIH-induced increases in cellular apoptotic rate also persisted after atorvastatin administration (*p* > 0.05, Figure 4C and 4D).

**Table 3:** Main and interaction effects of CIH and atorvastatin on apoptotic parameters.

	**BAX**		**Cleaved caspase-3**		**TUNEL positive cells**	
	**F**	***p***	**F**	***p***	**F**	***p***
CIH	140.10	<0.001	90.41	<0.001	12.17	<0.05
Atorvastatin	0.92	>0.05	1.05	>0.05	0.03	>0.05
Interaction	0.07	>0.05	0.12	>0.05	1.06	>0.05

Abbreviations: BAX: B-cell lymphoma 2-associated protein X; TUNEL: terminal deoxynucleotidyl transferase dUTP nick-end labeling.

## DISCUSSION

In this study, we created a mouse model that was exposed to 12 consecutive weeks of CIH to mimic pathophysiological changes that accompany OSA. Our results confirmed that CIH promoted myocardial oxidative stress and apoptosis. In addition, atorvastatin attenuated CIH-induced oxidative stress, but had no influence on apoptosis.

OSA, which has a high prevalence in general population [5], is correlated with increased risk of cardiovascular diseases, hypertension, and atherosclerosis [12]. OSA can increase the severity of cardiovascular events by inducing various pathophysiological changes, including endothelial dysfunction, systemic inflammation, oxidative stress, and apoptosis. Previous studies have demonstrated that OSA or CIH are closely associated with oxidative stress and apoptosis [13, 14]. Deng et al. [14] reported that CIH increased oxidative stress and apoptosis in the hippocampal neurons of mice. An *in vitro* study by Song et al. [15] showed that IH inhibited trophoblast cell motility and proliferation and induced excessive apoptosis by increasing endoplasmic reticulum stress. Consistent with these findings, our previous study [7] indicated that CIH promoted renal apoptosis. In the present study, we confirmed that myocardial oxidative stress and apoptosis were increased in CIH-treated mice. It is likely that treatments which inhibit CIH-induced oxidative stress and apoptosis might help reduce cardiovascular complications in OSA patients.

Independent of its lipid-regulating ability, atorvastatin has pleiotropic antithrombotic, anti-inflammatory, and antioxidant effects. Robust evidence indicates that atorvastatin may be helpful in treating various cardiovascular diseases, such as coronary heart disease, atherosclerosis, and hypertension. Tian and coworkers [3] found that atorvastatin improved cognitive disorders in a sepsis mouse model by mediating inflammatory cytokines, oxidative stress, and neuronal apoptosis in the hippocampus. Thassakorn et al. [16] reported that atorvastatin exerts antioxidant and anti-inflammatory effects in dogs with heart failure. An *in vitro* study by Wang et al. [17] reported that a combination of atorvastatin and caffeine suppressed proliferation and induced apoptotic death in prostate cancer cells. Li et al. [18] found that atorvastatin induced mitochondrial dysfunction and apoptosis in HepG2 cells. Finally, an *in vitro* study [19] showed that atorvastatin reduced aldosterone-induced vascular damage by inhibiting oxidative stress and inflammation in cultured chondrocytes.

Because CIH is closely associated with oxidative stress, inflammation, and apoptosis, the therapeutic effects of atorvastatin on animal models of OSA or CIH have received much attention. In 2013, Totoson et al. [10] reported that atorvastatin (10 mg/kg/d) administration reversed deleterious cardiovascular consequences, such as increased blood pressure, myocardial infarction hypersensitivity, and oxidative stress, and changes in endothelial function and carotid artery wall structure, in a CIH-induced rat model. Yuan and colleagues [11] found that atorvastatin (20 mg/kg/d) can attenuate TLR4/myeloid differentiation primary response protein 88-mediated inflammation and oxidative stress, thereby ameliorating CIH-induced myocardial injury. In a subsequent study, the same team found that atorvastatin (5 mg/kg/d) also reduced CIH-induced hippocampal neuronal injury in part through the TLR4 signaling pathway [9]. Ren and colleagues [20] reported that atorvastatin attenuated CIH-induced myocardial hypertrophy partly via the miR-31/PKC epsilon pathway *in vitro*. However, a multicenter randomized controlled clinical trial reported that, despite reduced blood pressure and lipid profile levels, 3 months of atorvastatin administration neither improved endothelial function nor reduced signs of atherosclerosis in OSA patients [21]. In the present study, we confirmed that atorvastatin attenuated myocardial oxidative stress after 12 consecutive weeks of CIH exposure. The antioxidant effects of atorvastatin included decreased MDA levels and increased SOD activity, T-AOC, and Nrf2 levels in mice subjected to CIH. However, in contrast to previous findings, atorvastatin did not affect CIH-induced myocardial apoptosis in our current study. Furthermore, the exact mechanisms underlying the effects of atorvastatin on CIH-induced pathophysiological changes remain unclear and require further investigation. Regarding hepatic toxicity of atorvastatin, although we observed slightly higher ALT and AST levels in atorvastatin-treated groups, those differences did not reach statistical significance. This relative lack of hepatic toxicity may be due at least in part to the lower dose we used in this study compared to those used in previous studies [10, 11, 22].

Several limitations of the present study should be considered when interpreting the results. Firstly, echocardiography, which is an important approach for evaluating cardiac structure and function, was not used here. Second, we did not examine potential molecular mechanisms that might explain the relationships between atorvastatin, CIH, and myocardial oxidative stress; additional studies are therefore required. Third, and similarly to previous animal studies [9–11, 23], only one atorvastatin dose was administered here, precluding our ability to examine dose-dependent effects of atorvastatin on IH-induced myocardial oxidative stress and hepatic toxicity.

In conclusion, we established a mouse model exposed to 12 consecutive weeks of CIH to more closely mimic OSA in this study. We found that atorvastatin treatment alleviated CIH-induced myocardial oxidative stress, but not apoptosis. These results provide new insights into the potential therapeutic benefits of atorvastatin treatment in OSA patients, especially those with cardiovascular comorbidities.

## MATERIALS AND METHODS

### Animal model and experimental groups

Forty-eight 7-week-old male C57BL/6 mice were purchased from the Chinese Academy of Science Laboratory Animals Center in Shanghai, China. All mice were kept in a departmental animal house on a 12:12-hour light-dark cycle with free access to water and food. The experiment was conducted for 12 consecutive weeks to create a more realistic CIH model. The body weight of each mouse was measured and recorded once per week. Mice were randomly assigned to one of four groups (*n* = 12 in each group): normoxia + vehicle, normoxia + atorvastatin, CIH + vehicle, or CIH + atorvastatin. The experimental procedure was approved (approval number 2018-015) by the Ethics Committee of Zhongshan Hospital, Xiamen University and was conducted according to the Guide for the Care and Use of Laboratory Animals [24].

### CIH exposure protocol

The CIH exposure protocol was performed as described in our previous studies [25, 26] with some modifications. Briefly, CIH-exposed mice were placed in a plexiglass chamber with one-way valves. Three gases (oxygen, nitrogen, and compressed air) which were controlled by a programmable instrument, flowed into the chamber constantly. The oxygen saturation in the chamber fluctuated from 21% to 6–8% and back in one two-minute hypoxia-reoxygenation cycle, for a total of 30 events per hour. Normoxia-exposed mice were placed in the same chamber but were exposed to the air in the room without manipulation. The CIH protocol was conducted from 09:00 to 17:00 daily for 12 consecutive weeks.

### Drug administration

Atorvastatin was purchased from Pfizer (Dalian, China) and dissolved in 10% ethyl alcohol in 200 μl tap water. From the 6^th^ week of the experiment on, atorvastatin (5 mg/kg/day) [9, 23] or vehicle (10% ethyl alcohol in 200 μl tap water) were administered to mice daily via oral gavage.

### Blood collection and tissue preparation

After the final CIH exposure, mice were deeply anesthetized and exsanguinated by cardiac puncture. After blood samples were collected and centrifuged, serum from the supernatant was stored for further analysis. The myocardial tissue of the left ventricle was excised and either stored at –80°C or fixed in buffered 10% formalin for future examination. Fresh myocardial tissues were homogenized with ice-cold radioimmunoprecipitation (RIPA) lysis buffer (Beyotime, Beijing, China). After centrifuging, protein concentrations in the supernatants were measured in a bicinchoninic acid protein assay (Beyotime, Beijing, China).

### Serum biochemical analysis

The following serum biochemical parameters were measured using a Hitachi 7020 automatic analyzer (Hitachi Co. Ltd., Tokyo, Japan): total cholesterol (TC), triglyceride (TG), high-density lipoprotein (HDL), low-density lipoprotein (LDL), alanine aminotransferase (ALT), and aspartate aminotransferase (AST).

### Oxidative stress detection

Malondialdehyde (MDA) levels, superoxide dismutase (SOD) activity, and total antioxidant capacity (T-AOC) were measured to evaluate antioxidant activity. All oxidative stress parameters were assayed in the homogenates of myocardial tissues according to the kit manufacturer’s instructions (Beyotime, Beijing, China). Briefly, MDA was evaluated spectrophotometrically by measuring amounts of thiobarbituric acid-reactive substances. SOD activity was detected using an assay kit which used a thiazole salt that produces a colored product in the presence of superoxide anions. T-AOC was analyzed using a colorimetric method based on the ferric-reducing ability of antioxidants. Absorbance values for MDA, SOD activity, and T-AOC assays were measured at wavelengths of 535 nm, 560 nm, and 593 nm, respectively.

### Western blotting analysis

Equal amounts of myocardial protein samples from each experimental group were separated by 10% sodium dodecylsulfate-polyacrylamide gel electrophoresis then transferred onto polyvinylidene difluoride membranes. After washing with TBS-0.1% Tween buffer and incubating with 5% non-fat dry skim milk, the membranes were incubated with the following primary antibodies at 4°C overnight: rabbit anti-B-cell lymphoma 2-associated protein X (BAX) (1:1000; Cell Signaling Technology [CST], Danvers, MA, USA), rabbit anti-cleaved caspase-3 (1:1000; CST), and rabbit anti-nuclear factor erythroid 2-related factor 2 (Nrf2) (1:2000; Abcam, Cambridge, MA, USA). After washing again with TBS-0.1% Tween, the membranes were incubated with horseradish peroxidase-conjugated secondary antibody for 1 hour at room temperature. The membranes were then treated with an enhanced chemiluminescence detection kit (Clarity™ Western ECL Substrate, Bio-Rad). β-actin was used as an internal control. Western blots were performed in triplicate for accuracy. Image J software (National Institutes of Health, Bethesda, MD, USA) was used to quantify band intensity.

### Hematoxylin-eosin (HE) and terminal deoxynucleotidyl transferase dUTP nick-end labeling (TUNEL) assay

After overnight fixation in formalin, myocardial tissues were dehydrated with alcohol, cleaned with xylene, and then embedded in paraffin. Five μm-thick slices were obtained and stained with HE. The TUNEL assay was carried out using the *in-situ* Cell Death Detection Kit, AP (Roche Diagnostics, Shanghai, China) according to the manufacturer’s instructions. TUNEL-positive cell numbers in the visual field were counted at ×400 magnification. The apoptotic rate was calculated as follows: TUNEL-positive cell number/total cell number × 100%. A Leica DM2500 microscope was used to visualize staining, and images were photographed at ×200 and ×400 magnification.

### Data analysis

SPSS software 23.0 (SPSS Inc., Chicago, IL, USA) and GraphPad Prism 5.0 (GraphPad Software, Inc., La Jolla, CA, USA) were used for statistical analysis and to make figures. Data are expressed as means ± SD. Two-way analysis of variance (ANOVA) followed by Dunnett post hoc tests were conducted for multiple comparisons between groups; main effects and interaction effects of week and CIH on bodyweight, as well as of CIH and atorvastatin on oxidative stress and apoptotic variables, were analyzed. A *p* value <0.05 was considered statistically significant.

### Ethics approval

This study was approved by the Ethics Committee of Zhongshan Hospital, Xiamen University (approval number 2018-015).

### Availability of data and material

All data generated or analyzed during this study are included in this published article.
